# Opioids Use and Abuse: Prescription Practice, Attitude, and Beliefs among Doctors of Karachi

**DOI:** 10.7759/cureus.5253

**Published:** 2019-07-27

**Authors:** Zainab Majid, Mahpara Tanveer, Sarrah Ali Asghar, Faryal Tahir, Areeba Minhaj, Hamza Aijaz Khan, Tehzeeb Sialvi, Syeda Hania Mahmood, Laila Tul Qadar, Fouzia Imtiaz

**Affiliations:** 1 Internal Medicine, Dow University of Health Sciences, Karachi, PAK; 2 Genetics, Dow University of Health Sciences, Karachi, PAK

**Keywords:** opioid abuse, prescription practice, karachi, opioid overdose, pakistan, pain management, opioid prescription

## Abstract

Background

Opioid analgesics, also known as narcotics, are medicinal drugs used primarily for the management of pain secondary to any type of cancer, severe injury or surgery. Due to the ease of availability, opioids are commonly abused. In 2015, reported deaths exceeded 33,000 Americans from opioid overdose. A survey in 2013 revealed nearly 1.6 million Pakistanis abusing prescription opioids for non-medical needs. Although commonly prescribed by primary care physicians, most of them are diffident to stand by all the recommended strategies to reduce the incidence of opioid abuse.

Methods

A cross-sectional study was conducted during the period of August through October 2018. A sample size of 365 was determined using a 95% confidence interval at a degree of precision of 5%. A 22-item questionnaire was given to doctors with at least two years practicing experience either from a private or a public healthcare setup. Doctors who had never prescribed opioids were excluded from the study. Out of the eligible participants, 15 refused to take part in the survey, and the co-operation rate was recorded as 95.8%. Collected data were analyzed using statistical package for social science (SPSS) version 22 for Windows. Frequencies, percentages, mean, standard deviation, and chi-square were used to explore the variables. The statistical significance level was considered at *p *< 0.5.

Results

Opioids were reported to be used mainly for treating acute pain (40.5%), chronic pain (24.7%) and both acute and chronic (34.8%). A minority of doctors (29%) screened their patients for opioid addiction. A significant association (*p* = 0.000) between the frequency of opioid prescription and prior screening for depression was determined. Surprisingly, only 23.2% clinicians frequently screened their patients for depression before prescribing opioids. The rate of counselling regarding drug tapering was found to be 71.6%. A majority, i.e., 88%, of the respondents anticipated the misuse of opioids they prescribe whereas 74% also held a belief that patients self-medicate their untreated pain. Participants reported addiction (54%) as the most common reason for abuse followed by the role of pharmaceutical companies (43%) and pharmacies (41%). About 80.2% clinicians believed that patients addicted to opioids could get well and return to their daily routine.

Conclusion

The rising opioid epidemic is a major concern for doctors prescribing opioids. Adaptation of medical school curricula and appropriate training can equip doctors for better management of patients requiring opioids. This includes the screening of patients using standard risk assessment tools for opioid abuse leading to a more controlled opioid prescription practice. Dissemination of these tools will boost doctors' confidence and may help in reducing morbidity and mortality from opioid abuse.

## Introduction

Opioids, usually termed as narcotics, act on opioid receptors to induce an effect analogous to morphine that causes reduction in pain. Primary medical uses of opioids include pain relief succeeding major injury or surgery, for the extreme pain of cancer and some doctors even prescribe them for chronic pain management in most cases [1-2]. Prescription opioids can lead to dependency and addiction. Dependence is expressed as experiencing withdrawal symptoms when not taking the drug, whereas addiction is a persistent mental disorder in which the person irresistibly seeks out drugs, although they produce harm [3]. The main cause of death in opioid addicts is overdosage which leads to the suppression of the respiratory centre in the brain. Every day, more than 115 people in the United States (US) confront death after overdosing on opioids. In 2015, more than 33,000 Americans died as a result of opioid overdose, including prescription opioids, heroin, and fentanyl, a potent synthetic drug [4]. According to the United Nations Office on Drugs and Crime, Pakistan is among the top 10 countries with the highest rate of addiction to opiates and opioids, with or without prescription [5]. As reported by the national survey on “Drug Use in Pakistan” conducted in 2013, 4.25 million people were found dependent on substances. Survey findings showed that nearly 1.6 million people reported misuse of prescription opioids for non-medical uses [6].

By 2009, prescription drugs surpassed motor vehicle accidents as a major cause of innocent deaths, with more people dying from prescription opioids than cocaine and heroin combined [7-8]. Although multiple factors bring about increase in injuries and deaths related to opioids, primary care physicians play a particularly vital role in promoting safe use of these products, as acute and chronic pain are amongst the routine clinical diagnosis in outpatient care, being frequently managed with prescription opioids [9-10]. Most physicians are diffident to stand by all the recommended strategies to reduce the incidence of opioid abuse. Inadequate rating of patient’s pain intensity by using a pain scale, misconception of the threats of prescribing fentanyl to opioid-naïve patients, hampered use of drug toxicology screens on patients either prior to or during opioid therapy, resistance to discontinue opioids if patients fail to meet the treatment goal can all promote opioid misuse [11].

According to a survey conducted on 1000 physicians in the US, only 25 percent of the physicians reported being ‘not at all’ or ‘only slightly concerned’ regarding the risk for deviation of opioid from licit to illicit market while this practice is frequent at all levels of pharmaceutical supply chain. In consonance with the same survey, only one-third (33 percent) of physicians believed that interpositions to minimize prescription opioid abuse had a moderate or major impact on the prevention of clinically appropriate access of patients to pain treatment [12]. With the increasing statistics of opioid abuse, it is important to highlight its prescription practices among doctors as well as their attitude and beliefs towards this issue. It is obligatory for providers and patients to discuss the possible risks of opioid, appraise alternative therapies, and if prescribing opioids seem pertinent, they should be given for fewer days and at lower dosages. In the current study, our primary objective was to determine the gaps between prescribing opioids for pain relief and considering possible risk factors for their abuse in the view of doctors practicing in Karachi. We also aimed to emphasize the relevance of their attitude and beliefs regarding opioid abuse. Thus, the secondary objective of this study was to fill these conspicuous gaps to minimize emergency visits with opioid overdose and related deaths. By improving the way opioids are prescribed, we can ensure that patients have access to safer and more effective chronic pain management while narrowing the burden of people who misuse, abuse, or overdose these potentially lethal drugs.

## Materials and methods

We conducted a cross-sectional study to describe the prescription practice, attitude, and belief regarding use and abuse of opioids among the doctors of Karachi, Pakistan. The study was conducted during the period of August through October 2018. The sample size using a 95% confidence interval and at a degree of precision of 5% was determined to be 350 subjects, calculated from OpenEpi.com.

The participants in our study comprised doctors with practicing experience of at least two years. We included doctors from the public as well as private hospital setups. Doctors without affiliations with other practice organizations or doctors with solo setups were also included. The exclusion criteria were doctors who had never prescribed opioids to patients. We also excluded doctors who have been out of practice for more than five years. A 22-item questionnaire that addressed the doctor’s prescription practice and attitude regarding general opioid use was prepared. A preliminary survey to 35 participants was carried out and based on the feedback, a few questions were modified, and some terms were made more relevant. The questionnaire was divided into three sections. A short demographic part inquired about the gender, type of work setup, duration of daily practicing hours and total years of practice. The first scale comprising of 14 questions observed participants' prescription practice such as the type of pain for which they usually prescribe opioids, factors that influence their decisions to prescribe, the frequency of Naloxone prescription and the counselling of patients regarding side effects. The second part contained eight questions to assess the attitude and beliefs of doctors regarding patients misusing the drug and their perception about the abuse among the general population. Doctors were also asked about the factors responsible for the rising abuse. 

The population-based sample was selected from different healthcare setups of Karachi using a convenience sampling. Before administering the questionnaire, the participants were explained the objective and benefits of the study and their verbal consent was obtained. Out of the 350 eligible doctors who were approached for this study, 22 subjects refused to participate; hence co-operation rate was recorded as 93.7%. The data collected were analyzed using Statistical Package for the Social Sciences (SPSS) version 22 for Windows. For categorical variables, frequencies and percentages were reported while for continuous variables, mean and standard deviation were reported. Chi-square was used to explore the variables. The level of statistical significance for all tests was considered at *p* < 0.5.

## Results

Our survey took place from August 2018 to November 2018. The convenience sampling strategy was used in the survey. A majority (*n *= 208, 63.4%) of respondents graduated in 2010 or later. More than half of respondents had a duration of practice of fewer than five years (*n *= 189, 57.6%), while 9.8% (*n *= 32) of respondents had a duration of practice greater than 10 and lesser than 15 years. Demographics are shown in Table 1.

**Table 1:** Demographics *Others: include group practices, health maintenance organizations (HMOs), locum tenens, etc.

VARIABLES	FREQUENCY (%)	
Year of graduation	
1979 or earlier	4 (1.2)
1980-1989	29 (8.8)
1990-1999	34 (10.4)
2000-2009	53 (16.3)
2010-later	208 (63.4)
Practice mode	
Private solo	27 (8.2)
Private hospital	65 (19.8)
Government hospital	232 (70.7)
Community health center	2 (0.6)
*Others	2 (0.6)
Working hours	
Less than 6 hours	29 (8.8)
6-8 hours	142 (43.3)
9-12 hours	58 (17.7)
More than 12 hours	99 (30.2)
Duration of practice	
Less than 5 years	189 (57.6)
Less than 10 years	72 (22.0)
Less than 15 years	32 (9.8)
More than 15 years	35 (10.7)

Most opioids were used for treating acute pain (*n* = 133, 40.5%), whereas 24.7% (*n *= 81) participants prescribed opioids to treat chronic pain and 34.8% (*n *= 114) used opioids to treat both acute and chronic pain. However, no evident association was found between the frequency of opioid prescription and reasons for which the respondent mostly prescribed opioids, i.e. for treating acute pain, chronic pain or both (*p *= 0.205).

A minority (*n *= 95, 29%) of the respondents screened their patient's urine or serum for opioid addiction before prescribing opioids while the majority (*n* = 233, 71.0%) did not screen their patients. There is a significant association between the frequency of opioids prescribed and screening of the patients before prescribing opioids (*p *= 0.000).

We asked participants about the frequency with which they screen their patients for depression prior to prescribing opioids. Astonishingly, only 33.2% (*n *= 109) doctors regularly performed screening for depression, whereas 27% (*n *= 88) evaluated sometimes, 26.5% (*n *= 87) rarely and 13.4% (*n *= 44) never took into account the depression of patients.

When asked whether the doctors counselled their patients about the drug tapering before prescribing opioids, 71.6% (*n *= 235) of participants responded positively. There was a significant association (*p *= 0.017) among respondents who advised their patients about the tapering of drugs and frequency of prescription. We also inquired the doctors regarding the frequency of prescribing Naloxone and found that only 4% (*n *= 13) of doctors always prescribed Naloxone to patients receiving opioids, while 35.1% (*n *= 115) of respondents never prescribed Naloxone.

We applied the chi-square test to find out the association between the frequency of opioids prescribed and practice setting of the participants (*p *= 0.615), and the relation between gender of participants and frequency of opioids prescription, which is insignificant (*p *= 0.756).

When asked whether the doctors feel if there is a lack of a safe and effective alternative to opioids, more than half (*n *= 227, 69.2%) responded positively. A majority (*n *= 263, 80.2%) of the doctors believed that patients addicted to opioid could get well and return to their healthy lives. Moreover, 133 (40.5%) doctors believed that there are aggressive marketing and promotion of pain medications without adequate knowledge of their safety and effectiveness.

According to 59.8% (*n *= 196) respondents, there was no gender predilection of patients to opioid prescription, although 23.2% (*n *= 76) believed that female patients are prescribed most opioids, while 17.1% (*n *= 56) believed that males were receiving more of the drug. Only 7.6% of participants believed that patient satisfaction did not influence their opioid prescription. A vast majority (*n *= 270, 82.4%) of respondents considered opioid therapy after other therapies failed.

**Figure 1:**
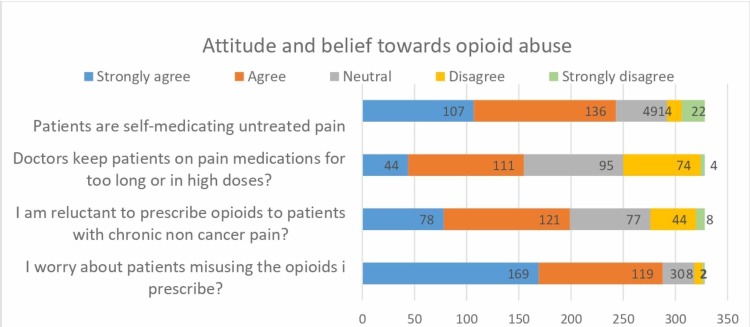
Respondents attitude and belief towards opioid abuse

**Figure 2:**
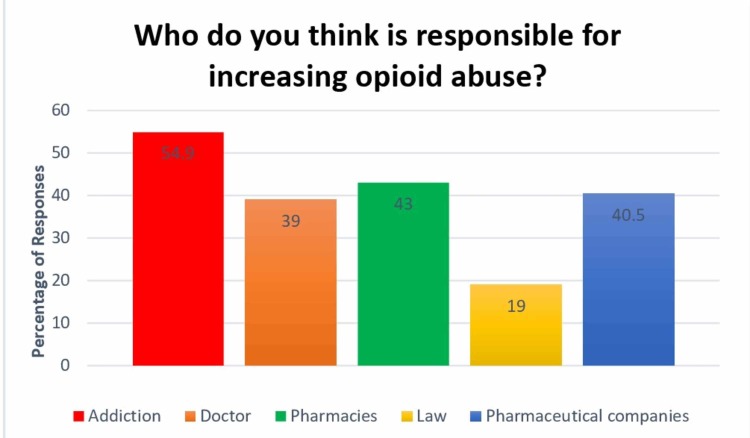
Responses on who doctors think is responsible for increasing opioid use

More than half of the participants (87.8%, *n *= 288) anticipated the misuse of opioids they prescribe, while only 0.6% (*n *= 2) did not suspect any misuse by their patients. This might be the reason for scattered data regarding reluctance for prescribing opioids. Additionally, it is strongly believed that patients are self-medicating their untreated pain (32.6%, *n *= 107), as shown in Figure 1. The study highlights the strong opinions of participants that addiction is the most common reason for abuse (54.9%, *n *= 180), but not much lagging behind are pharmaceutical companies (40.5%, *n *= 133) and pharmacies (43%, *n *= 140; Figure 2).

## Discussion

The study provides useful insights into prescription practices and perception of opioid abuse among doctors of Karachi. We found that the physicians’ prescription practices, in terms of assessing the patient before administering opioid therapy are moderately reliable. Our data shows most of the doctors assess the level of pain using pain scale. Pain score is a major step in the “universal precautions” approach to assessing the risk of opioid abuse among chronic pain patients as proposed by Gourlay et al. [13]. Other than pain, our study assessed other factors from universal precautions like psychological analysis, an appropriate trial of opioid therapy, and urine or serum screening.

Untreated psychiatric disorders are one of the risk factors for opioid abuse [14]. The prevalence of major depressive disorder among other psychiatric disorders in chronic pain patients is high [15]. We found that only a minority of the doctors screened their patients for depression before prescribing opioids. This is particularly alarming as in a review of the relation between depression and opioid use, abuse, and addiction by Sullivan MD, it was established that depressed patients may tend to overuse opioids because they use them to treat insomnia and stress [16]. However, we also found that a majority of doctors counsel their patients about opioid side effects which include physical dependence and tolerance [17]. Therefore, it can be suggested that doctors should take this counselling further ahead by adding depression screening as part of their assessment. 

We found that a clear majority of the respondents are reluctant to prescribe opioids for chronic non-cancer pain. This may be due to the gap in doctors’ knowledge regarding risk factor assessment tools for opioid addiction. In a survey, most physicians rated their knowledge/comfort of treatment/management of opioid dependence as being low [18]. As such, a lot of surveys including various specialties like family physicians, surgical interns, obstetrician-gynecologists call for better opioid prescribing training and education [19-21]. In another study where the intervention group received education on the assessment of the risk of opioid misuse, the rate of eagerness to apply risk assessment increased markedly [22]. By adapting curricula to address the rising opioid epidemic, medical schools have the potential to ensure that future physicians can effectively recognize the signs, symptoms, and risks of opioid abuse and improve patient outcomes [23].

Our data shows that most physicians believed individual-oriented factor of addiction as the cause of increasing opioid abuse, followed by pharmaceutical companies. Previous surveys including primary care physicians also report similar findings [24-25]. Our results show that 40.5% of the doctors believed that there is “a lot” of marketing and promotion of pain medications without adequate knowledge of their safety and effectiveness and only 9.8% believed that there was “not at all” marketing and promotion. Hendricks et al. comparably report that approximately half (50%) of the respondents endorsed causal attributions related to the pharmaceutical companies, i.e., marketing without inadequate explanation of addiction risks on the medication labels (54%) and promotion of these medications without adequate knowledge of their effectiveness and safety (46%) [25]. This maybe due to the drug reps, hired by these companies, who increase drug sales by influencing the physicians [26].

Regarding the doctors' attitudes and beliefs on opioid therapy, we found out that majority (80.2%) of the doctors believed that patients addicted to opioids can get well and return to their normal lives with treatment. This belief contrasts with the Prescription Opioid Addiction Treatment Study (POATS), the largest clinical trial yet conducted on prescription opioid addicts by the National Drug Abuse Treatment Clinical Trials Network, which showed that weekly medical management and counselling render no overall benefit in discontinuing opioids addiction; very little (7%) of the study participants responded positively and returned back to their opioid-free lives during the Phase-1 i.e. (Week-1 taper) of this study [27].

We also found that when asked if the doctors feel that there is a lack of safe and effective alternative to opioids, more than half (69.2%) of doctors responded positively, suggesting that the options for pain management are limited. This finding contrasts with the result of a previous survey held to evaluate the attitudes of primary care physicians (PCPs) and patients regarding opioid use, which revealed the belief of PCPs that the health plan had introduced several initiatives to reduce and better manage the use of opioids [28]. However, in the same study, the patients responded that pain medications (particularly opioids) were the only option available and they were not satisfied with other modes of treatment. This dissatisfaction of the patients also influenced the doctors' prescription practice; PCPs stated that they were unable to convince the patients on switching to other modalities of treatment for chronic pain management and ended up prescribing opioids.

There are several limitations in our study which need to be considered. Firstly, we did not ask the doctors if they use any standard tool like Screener to Predict Opioid Misuse in Chronic Pain Patients (SOAPP-R) to assess at-risk patients for opioid abuse. Secondly, most of the respondents prescribed opioids occasionally or had been practising for less than five years; therefore, it can be understood that their prescription practice may not be long enough to have better perception of the rising epidemic of opioid abuse. Thirdly, most of the doctors practised in government hospitals so the data maybe inadequate to represent private hospital practitioners.

## Conclusions

With the increasing rate of opioid abuse, future propositions and efforts should be directed towards the reduction of the gap between opioid abuse and its prescription practice. Appropriate steps must be taken to increase awareness among doctors regarding all the possible risk factors for opioid abuse. Updating medical curricula and using opioid abuse risk assessment tools can decrease the rising incidence of mortality secondary to opioid overdose.
